# Identification of Site-Specific Stroke Biomarker Candidates by Laser Capture Microdissection and Labeled Reference Peptide

**DOI:** 10.3390/ijms160613427

**Published:** 2015-06-11

**Authors:** Tingting Lian, Daixin Qu, Xu Zhao, Lixia Yu, Bing Gao

**Affiliations:** School of Bioscience and Bioengineering, South China University of Technology, Higher Education Mega Center, Guangzhou 510006, China; E-Mails: l.tt04@mail.scut.edu.cn (T.L.); qu.daixin@mail.scut.edu.cn (D.Q.); rogerxu8911@gmail.com (X.Z.); yulixia2015@gmail.com (L.Y.)

**Keywords:** laser capture microdissection (LCM), labeled reference peptide (LRP), blood–brain barrier (BBB), stroke, biomarker, multiple reaction monitoring (MRM)

## Abstract

The search to date for accurate protein biomarkers in acute ischemic stroke has taken into consideration the stage and/or the size of infarction, but has not accounted for the site of stroke. In the present study, multiple reaction monitoring using labeled reference peptide (LRP) following laser capture microdissection (LCM) is used to identify site-specific protein biomarker candidates. In middle cerebral artery occlusion (MCAO) rat models, both intact and infarcted brain tissue was collected by LCM, followed by on-film digestion and semi-quantification using triple-quadrupole mass spectrometry. Thirty-four unique peptides were detected for the verification of 12 proteins in both tissue homogenates and LCM-captured samples. Six insoluble proteins, including neurofilament light polypeptide (NEFL), alpha-internexin (INA), microtubule-associated protein 2 (MAP2), myelin basic protein (MBP), myelin proteolipid protein (PLP) and 2′,3′-cyclic-nucleotide 3′-phosphodiesterase (CNP), were found to be site-specific. Soluble proteins, such as neuron-specific enolase (NSE) and ubiquitin carboxyl-terminal hydrolase isozyme L1 (UCHL1), and some insoluble proteins, including neurofilament heavy polypeptide (NEFH), glial fibrillary acidic protein (GFAP), microtubule-associated protein tau (MAPT) and tubulin β-3 chain (TUBB3), were found to be evenly distributed in the brain. Therefore, we conclude that some insoluble protein biomarkers for stroke are site-specific, and would make excellent candidates for the design and analysis of relevant clinical studies in the future.

## 1. Introduction

Numerous studies have focused on blood biomarker discovery for ischemic stroke and a variety of protein biomarker candidates have been identified [1]. However, the accuracy and reproducibility of these biomarker candidates still need to be improved. The release of glial and neuronal proteins into the blood varies according to the complexity and heterogeneity of lesions after a stroke. The plasmatic levels of protein biomarkers have been correlated to age, genetic background, gender and predisposing factors, such as infection and inflammation, as well as the stage and/or the size of infarction [2,3,4,5]. However, the site of infarction has not been considered as an important indicator in previous clinical studies [1,6], and more effort is required to identify site-specific protein biomarker candidates for acute ischemic stroke.

Accurate measurement of protein levels in infarcted tissue is a challenge because the weight of tissue sample cannot be used for normalization due to its heterogeneous composition. Laser capture microdissection (LCM) provides an approach to make normalization possible based on the area of tissue sample, and thus it could become a new assay to assess protein distribution and stability in infarcted brain tissues. Since 1996, traditional tissue cutting has been replaced by LCM for procuring pure cell populations in a quick and automated manner [7]. In 2014, Subramanian *et al.* collected the fluorescently labeled neurons from sections encompassing the rostrocaudal extent of the rostral ventrolateral portion of the medulla by LCM [8]. In 2014, genetically labeled neurons from the drosophila melanogaster brain were isolated by LCM, and the catapulted tissue patches were collected on a reversed phase column, and analyzed using an on-column extraction (OCE) that was directly coupled with liquid chromatography–multistage mass spectrometry (LC-MS(*n*)) [9].

Targeted proteins from LCM-dissected homogeneous cell populations can be quantitatively analyzed by multiple reaction monitoring (MRM) using stable isotope dilution (SID). Bagnato *et al.*, measured cytokines and growth factors in coronary arteries by MRM assay. Four ^13^C/^15^N-labeled peptides for cytokines/growth factors were spiked into LCM-procured samples to complement the shotgun proteomics approaches for low abundance protein identification [10]. In 2011, Guzel *et al.*, measured preeclampsia-related calcyclin peptides using peptides with an extra glycine inserted in the endogenous sequence as an internal standard instead of the heavy labeling method for MRM assay [11]. Attempts have been made to quantify proteins of interest at a low cost. In 2011, Zhang *et al.* developed a labeled reference peptide (LRP) method that uses a single labeled peptide as a reference standard for all targeted peptides and measures peptides based on their un-normalized peak areas for detected MRM transitions [12].

**Figure 1:**
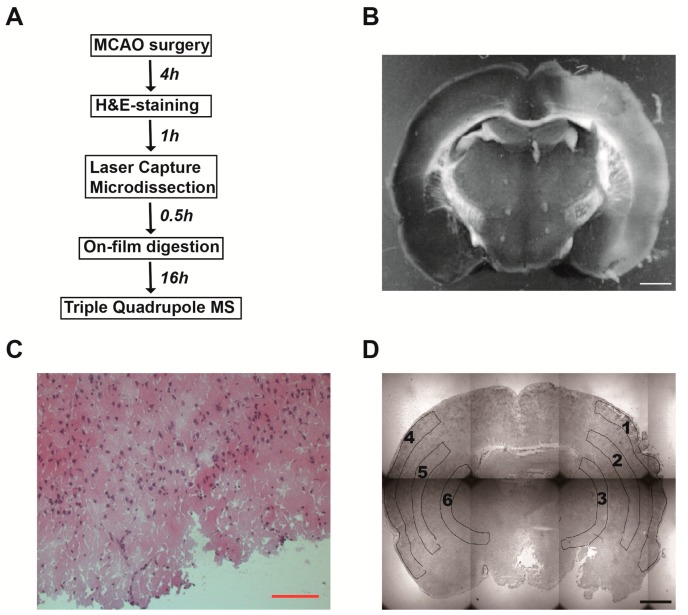
The focal cerebral ischemic model. (**A**) Scheme of sample collection, processing and analysis; (**B**) The extent of cerebral infarct was shown by TTC (2,3,5-triphenyltetrazolium chloride) staining. Scale bar: 1 mm; (**C**) Representative hematoxylin and eosin (H&E)-stained mouse brain sections at 4 h after the occlusion of middle cerebral artery. Scale bar: 100 µm; (**D**) Laser capture microdissection (LCM) of intact and infarcted tissue in the cerebral cortex. Microscopic findings of focal ischemic tissue in the right cerebral cortex and normal tissue in the left cerebral cortex. Coronally cut fresh sections were stained with H&E and shown at a low magnification. Zones 1–3 of ischemic brain tissue supplied by right middle cerebral artery were collected by LCM. The corresponding areas (zones 4–6) in the left cerebral cortex were also collected as a control. The size of each area was measured. Scale bar: 1 mm.

As indicated above, LCM followed by MRM using LRP (LCM–LRP) is an established tool for screening site-specific protein biomarkers in both intact and infarcted tissue. By using LCM–LRP, we can normalize the level of targeted protein by the capture area of LCM that remains the same for infarcted tissue and intact tissue. The procedure used in the present study is summarized in Figure 1A. We procured brain tissue samples from middle cerebral artery occlusion (MCAO) rat model brain by LCM and measured proteins of interest by triple-quadrupole mass spectrometry using the LRP method. Samples included infarcted brain tissue from zones 1–3 and intact brain tissue from zones 4–6 (Figure 1D). Thirty-four unique peptides of 12 proteins are verified and semi-quantified in both brain tissue homogenates and LCM-procured samples. Reference peptides, with or without ^13^C and ^15^N labeling, and maltose-binding protein (mbp) are added to all samples for quality control purposes. Peak area ratios of each unique peptide in zones 1–3 and 4–6 were measured and compared, the distribution of the corresponding protein evaluated, and site-specific protein biomarkers identified.

## 2. Results and Discussion

### 2.1. The Focal Cerebral Ischemic Model in Rats

Most human ischemic strokes are caused by occlusion of the middle cerebral artery (MCA), and thus intraluminal MCA occlusion model in rat is the most common model of focal cerebral ischemia. In the present study, we established a permanent focal cerebral ischemia model by inserting a monofilament into the internal carotid artery (ICA) to block blood flow to the MCA for 4 h, and then grade neurological deficits of these MCAO animals by Longa neurologic score [13]. All MCAO rats in this study are scored 2.0 (circulating to the left) and considered as successful focal cerebral ischemic models (supplementary video 1).

The area of infarction after occlusion of the MCA is identified by 2,3,5-triphenyltetrazolium chloride (TTC) staining, which makes infarcted regions white (Figure 1B). Size and location of the infarction were determined by hematoxylin and eosin (H&E) staining (Figure 1C). Condensed, dead neurons with eosinophilic cytoplasm and/or pyknosis are scattered throughout the spongy edematous necrotic neutrophil. The swollen astrocytic processes are arranged in parallel. There is evidence of infiltration by monocytes and macrophages, as well as their mitotic figures.

### 2.2. Laser Capture Microdissection (LCM) of Intact and Infarcted Tissue in the Cerebral Cortex

**Figure 2:**
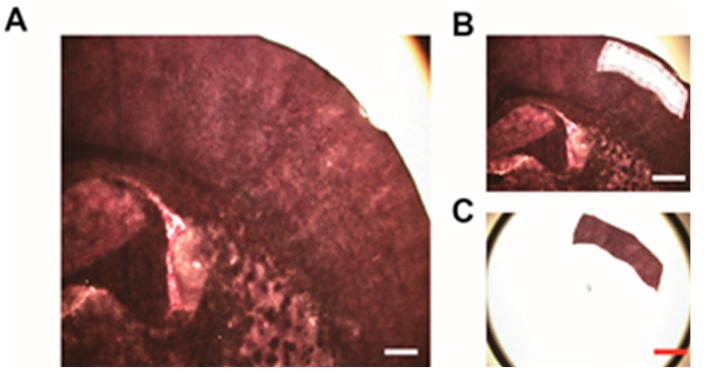
Sample collection by laser capture microdissection (LCM). (**A**) Infarcted tissue in the cerebral cortex before LCM. Scale bar: 0.5 mm; (**B**) Infarcted tissue in the cerebral cortex after LCM. Scale bar: 1.0 mm; (**C**) LCM-procured tissue samples. Scale bar: 1.0 mm.

To quantitatively analyze protein levels in the infarct region, both intact and infarcted tissue on the same brain slice were collected by LCM at a low magnification (Figure 1D and Figure 2). Coronally cut fresh sections were stained with H&E and focal infarct areas in the right cerebral cortex were identified. Based on both TTC and H&E staining, the cerebral cortex was divided into three zones, with the outer layer designated as zone 1, the inner layer as zone 3, and the middle layer as zone 2. These zones are supplied by the right middle cerebral artery before occlusion. The corresponding zones (4–6) in the left cerebral cortex were also collected as a control. The total captured area of each zone was 10 mm^2^ (Table 1, *n* = 12).

**Table 1:** Area of brain tissue obtained by laser capture microdissection (LCM).

Sample Size	Zone 1	Zone 2	Zone 3	Zone 4	Zone 5	Zone 6
LCM Area (mm^2^)	10.36 ± 0.07	9.96 ± 0.08	9.67 ± 0.05	10.47 ± 0.07	9.87 ± 0.07	9.71 ± 0.07

### 2.3. Semi-Quantification of Quality Control Protein by Labeled Reference Peptide (LRP) Analysis

**Figure 3:**
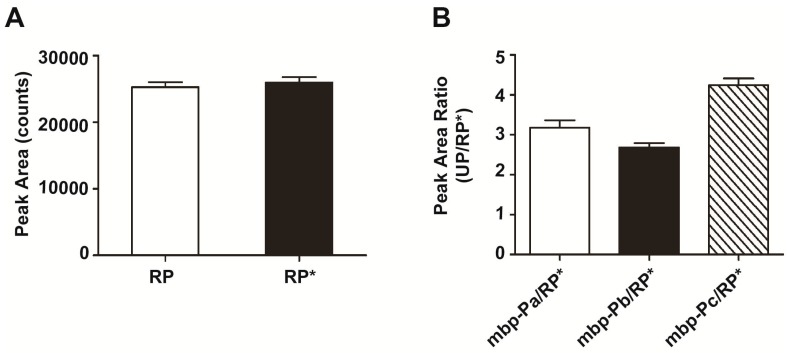
Semi-quantitative analysis of LCM-procured samples by triple-quadrupole mass spectrometry. (**A**) One ng of ^13^C and ^15^N labeled reference peptide (APGLTQALNTK*) and 1 ng of unlabeled reference peptide (APGLTQALNTK) were added to all samples as a quality control (*n* = 72); (**B**) A total of 150 ng of maltose-binding protein (mbp) was added to all samples and the peak areas of three mbp unique peptides (mbp-Pa, DVGVDNAGAK; mbp-Pb, AGLTFLVDLIK; mbp-Pc, VNYGVTVLPTFK) were measured (*n* = 72). The peak area ratio of each mbp unique peptide to RP* is shown. RP, unlabeled reference peptide; RP*, labeled reference peptide.

One ng of the unique peptide (APGLTQALNTK*) with ^13^C and ^15^N labeling at the C-terminal lysine and 1 ng of the same peptide without labeling were added to all samples as reference peptides (RP). The peak area of unlabeled RP is 25,237 ± 766.7 (counts) and that of labeled RP (RP*) is 25,952 ± 815.9 (Figure 3A, *n* = 72). The peak area ratio of RP *versus* RP* is 0.9959 ± 0.02496 (Table 2, *n* = 72), as compared to a theoretical value of 1.0000.

**Table 2:** Quantification of unlabeled reference peptide (RP), labeled reference peptide (RP*) and maltose-binding protein (mbp) unique peptides.

Peptide	Sequence	Molecular Weight (Da)	Peak Area	Peak Area Ratio (RP*)	Theoretical Amount (ng)
RP	APGLTQALNTK	1113.261	25,237 ± 766.7	0.9959 ± 0.02496	1
RP*	APGLTQALNTK*	1121.261	25,952 ± 815.9	1.0000	1
mbp-Pa	DVGVDNAGAK	944.979	77,202 ± 5126	3.182 ± 0.1818	3.336641
mbp-Pb	AGLTFLVDLIK	1189.442	65,117 ± 3118	2.687 ± 0.1039	4.199819
mbp-Pc	VNYGVTVLPTFK	1337.562	103,733 ± 5828	4.242 ± 0.1669	4.722818

A total of 150 ng of maltose-binding protein (mbp, M.W. 42,481.902 Da) was added to all samples and the peak areas of three mbp unique peptides (mbp-Pa: DVGVDNAGAK, mbp-Pb: AGLTFLVDLIK, mbp-Pc: VNYGVTVLPTFK) were measured (Table 2). The peak area ratio of each mbp unique peptide (UP) *versus* RP* (UP/RP*) indicates the amount of that unique peptide estimated by LRP analysis. These values and the corresponding theoretical amounts for each mbp peptide, equal to 150 ng × (M.W. of mbp peptide/M.W. of MBP), are shown in Table 2. For example, the estimated amount of mbp-Pa is 3.182 ± 0.1818 ng, a value close to its theoretical amount of 3.336641 ng. The standard errors of peak area ratios of mbp-Pa, -Pb and -Pc are all small enough to allow a reliable semi-quantitative analysis of mbp protein (Table 2, Figure 3B, *n* = 72).

### 2.4. Distribution of Neurofilament- and Microtubule-Related Proteins in both Intact and Infarcted Brain Tissues

After focal ischemia, blood–brain barrier (BBB) permeability increases at the site of infarction or the distant region of the ipsilateral cerebrum, allowing blood proteins such as albumin to cross the BBB and enter the interstitial space, while glial and neuronal proteins enter the blood [14]. Biomarker protein candidates, including glial fibrillary acidic protein (GFAP), myelin basic protein (MBP) and neuron-specific enolase (NSE) are released into the blood through the disrupted blood–brain barrier. In this study, LCM-captured samples from zones 4–6 in intact brain tissues and zones 1–3 in infarcted brain tissues were analyzed by LRP using a triple-quadrupole mass spectrometer. Four neurofilament proteins, including neurofilament light polypeptide (NEFL), neurofilament heavy polypeptide (NEFH), alpha-internexin (INA) and glial fibrillary acidic protein (GFAP), and three microtubule proteins, including microtubule-associated protein 2 (MAP2), microtubule-associated protein tau (MAPT) and tubulin beta-3 chain (TUBB3), were verified by their unique peptides (Table S1).

**Figure 4:**
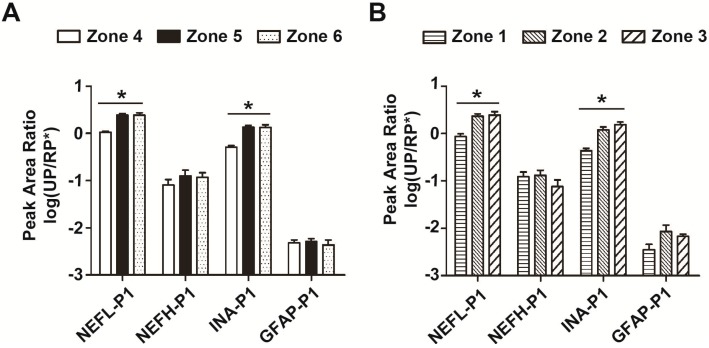

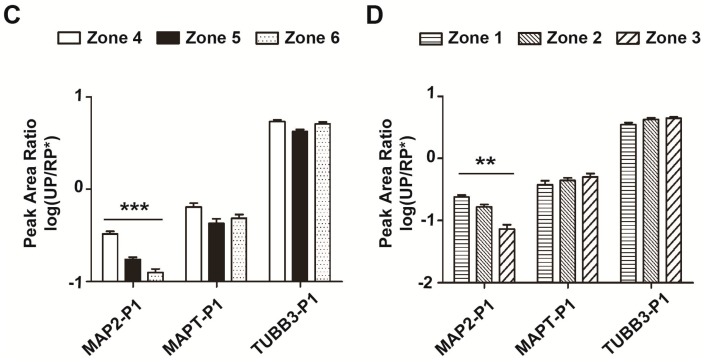
Distribution of neurofilament and microtubule peptides/proteins in both intact and infarcted brain tissues. (**A**) Proteins in zones 4–6 of intact brain tissue were collected by LCM, 10 mm^2^ for each area, and then analyzed by a triple-quadrupole mass spectrometer. Four neurofilament proteins were verified and semi-quantified by LCM–LRP. Each protein was verified by 2–3 unique peptides and quantified by the peak area. The unique peptides with the greatest peak area are shown. Peak area ratios of unique peptides to RP* were calculated (UP/RP*) (*n* = 12); (**B**) Four neurofilament proteins in zones 1–3 of infarcted brain tissue were collected by LCM, and then the peak area ratios of 4 unique peptides to RP* were determined (*n* = 12); (**C**) There were 3 microtubule proteins from zones 4–6 of intact brain tissues verified and semi-quantified by LCM–LRP. Each protein was verified by 2–3 unique peptides and quantified by the peak area. The unique peptides with the greatest peak area are shown. Peak area ratios of unique peptides to RP* were calculated (UP/RP*) (*n* = 12); (**D**) Three microtubule proteins in zones 1–3 of infarcted brain tissue are collected by LCM, and then the peak area ratios of 3 unique peptides to RP* were determined (*n* = 12). Unique peptide 1 for neurofilament light polypeptide (NEFL-P1), FTVLTESAAK; unique peptide 1 for neurofilament heavy polypeptide (NEFH-P1), FAQEAEAAR; unique peptide 1 for α-internexin (INA-P1), AQLEEASSAR; unique peptide 1 for glial fibrillary acidic protein (GFAP-P1), ALAAELNQLR; unique peptide 1 for microtubule-associated protein 2 (MAP2-P1), GLSSVPEVAEVETTTK; unique peptide 1 for microtubule-associated protein tau (MAPT-P1), TPSLPTPPTR; unique peptide 1 for tubulin beta-3 chain (TUBB3-P1), YLTVATVFR. * *p* < 0.05; ** *p* < 0.01; *** *p* < 0.001.

Unique peptides with the greatest peak area for each protein are highlighted in Table S1 and used for semi-quantification of their corresponding proteins. GFAP is a monomeric intermediate filament protein in astrocytes and its protein level in the serum reaches a maximum between day 2 and 4 after acute ischemic stroke [15]. Serum GFAP is detectable within 6 h after acute intracerebral hemorrhage (ICH) with a sensitivity of 0.79 and a specificity of 0.98 [16]. By LCM–LRP, we found that GFAP is evenly distributed in zones 4–6 or zones 1–3 (Figure 4A,B), which suggests that the plasma level of GFAP is not related to the site of stroke. NEFH is a major component of the intermediate filaments in neurons, and it plays an important role in mature axons [17]. Our results indicate that NEFH is also evenly distributed in both intact and infarcted brain tissues (Figure 4A,B), and thus not site-specific. INA is the counterpart of GFAP in neurons and it may form an intermediate filament network with or without the involvement of NEFL [18]. INA is believed to play a role in axonal outgrowth. Lower levels of INA and NEFL were found in zone 4 than zones 5–6 , which indicates that more INA and NEFL is present in the inner layer of cerebral cortex. MAPs and tubulin are major components of microtubules, with MAP2 being found mostly in dendrites and MAPT in the axon [19,20]. In the present study, more MAP2 was found in zone 4 than zones 5 and 6, but MAPT and TUBB3 were evenly distributed in all areas of intact brain tissues (Figure 4C, Table S2, *n* = 12), and the distribution pattern of these biomarker candidates was similar in zones 1–3 (Figure 4D, Table S2, *n* = 12).

### 2.5. Site-Specificity of Myelin-Related Proteins in Intact and Infarcted Brain Tissues

**Figure 5:**
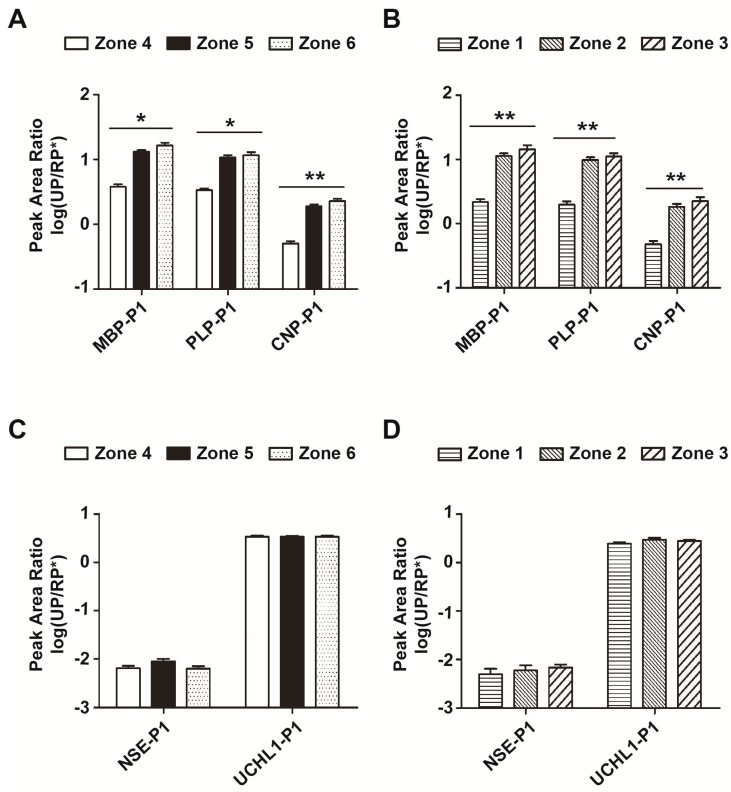
Distribution of myelin proteins and other biomarker candidates in intact and infarcted brain tissue. (**A**) Three myelin proteins from zones 4–6 of intact brain tissues were verified and semi-quantified by LCM–LRP. Each protein was verified by 2–4 unique peptides and quantified by the peak area. Peak area ratios of unique peptides to RP* were calculated (UP/RP*) (*n* = 12); (**B**) Three unique myelin proteins in zones 1–3 of infarcted brain tissue were collected by LCM, and then the peak area ratios of 3 unique peptides to RP* were calculated (UP/RP*) (*n* = 12); (**C**) Two protein biomarker candidates from zones 4–6 were verified by 2–3 unique peptides for each and quantified by the peak area. Peak area ratios of unique peptides to RP* were calculated (UP/RP*) (*n* = 12); (**D**) The peak area ratios of NSE-P1 and UCHL1-P1 (UP/RP*) in zones 1–3 were calculated (*n* = 12). Unique peptide 1 for myelin basic protein (MBP-P1), DTGILDSIGR; unique peptide 1 for myelin proteolipid protein (PLP-P1), TSASIGSLCADAR; unique peptide 1 for 2′,3′-cyclic-nucleotide 3′-phosphodiesterase (CNP-P1), AIFTGYYGK; unique peptide 1 for neuron-specific enolase (NSE-P1), LGAEVYHTLK; unique peptide 1 for ubiquitin carboxyl-terminal hydrolase isozyme L1 (UCHL1-P1), FSAVALCK. * *p* < 0.05; ** *p* < 0.01.

Myelin basic protein (MBP), myelin proteolipid protein (PLP) and 2′,3′-cyclic-nucleotide 3′-phosphodiesterase (CNP) are major constituents of the CNS myelin [21,22]. Hall *et al.* found that 39% of patients with acute ischemic stroke had positive test results for blood MBP on admission [6]. Jauch *et al.* found that the concentration of MBP increased at 24 h after treatment [23]. Our results show that MBP, PLP and CNP were present more abundantly in zones 5 and 6 than in zone 4 (Figure 5A, Table S2, *n* = 12), and also more abundant in zones 2 and 3 than in zone 1 (Figure 5B, Table S2, *n* = 12). In other words, MBP, PLP and CNP are all site-specific (Figure 5A,B). Therefore, the location of lesions may have to be considered for future clinical studies.

Neuron-specific enolase (NSE) is a glycolytic enzyme in neurons, also known as gamma-enolase or enolase 2 (ENO2). Ubiquitin carboxyl-terminal hydrolase isozyme L1 (UCHL1) is abundantly present in all neurons [24]. Both NSE and UCHL1 are soluble proteins in the cytoplasm that are not thought to be site-specific, and our results confirm that they are evenly distributed in zones 4–6 and 1–3 (Figure 5C,D).

### 2.6. Abundance and Stability Analysis of Protein Biomarker Candidates by LCM–LRP

**Figure 6:**
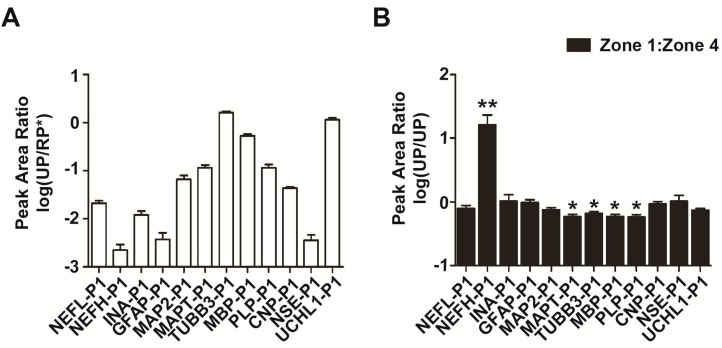
Abundance and stability analysis of protein biomarker candidates by LCM–LRP. (**A**) Twelve protein biomarker candidates in brain tissue homogenates were verified and semi-quantified by triple-quadrupole mass spectrometry. The abundance of each protein was estimated by the peak area ratio of its corresponding unique peptide (*n* = 12); (**B**) Peak areas of 12 unique peptides in zone 1 were compared to those in zone 4 (1:4), and the relative peak area ratios (UP/UP) are shown (*n* = 12). * *p* < 0.05; ** *p* < 0.01.

Peak area ratios (UP/RP*) of 12 unique peptides in 5 µg of brain tissue homogenates were shown to estimate the abundance of protein biomarker candidates (Figure 6A, *n* = 4). By LCM–LRP analysis, we can also estimate the stability of proteins in the center of infarcted regions by the peak area ratios for zone 1 to zone 4 (UP/UP, 1:4) (Figure 6B, *n* = 12). Peak area ratios (UP/RP*) of unique peptides of NEFL, INA, GFAP, MAP2, CNP, NSE and UCHL1 in zone 1 were similar to those in zone 4 (Figure 4 and Figure 5, *n* = 12), and their peak area ratios (UP/UP, 1:4) were close to 1.0 (Figure 6B, *n* = 12), which suggests that these potential biomarkers are stable in the center of ischemic brain tissue. The peak area ratios (UP/UP, 1:4) of unique peptides of MAPT, TUBB3, MBP and PLP were significantly lower than 1.0 (Figure 6B, *p* < 0.05), which indicates that these proteins may be degraded at 4 h after ischemia and are not ideal protein biomarker candidates. The peak area ratio (UP/UP, 1:4) of NEFH unique peptide 1 is 16.275 ± 6.982 (Figure 6B, *p* < 0.01, *n* = 12), which suggests that NEFH protein concentration is increased in the early stage of ischemia (≤4 h).

## 3. Experimental Section

### 3.1. Materials

Chloral hydrate was purchased from KeLong Chemical (Chengdu, China). Deionized water was purified by a Millipore Advantage A10 system (Millipore, Bedford, MA, USA). An amount of 90% ethanol (HPLC grade) was purchased from Thermo Fisher Scientific (Rockford, IL, USA). Solutions of 70% (*v*/*v*) ethanol were prepared with purified H_2_O. Xylene was purchased from Sigma Aldrich (St. Louis, MO, USA). Mayer’s hematoxylin solution and wosin Y solution were purchased from Merck (Whitehouse Station, NJ, USA). The 2,3,5-triphenytetrazolium chloride (TTC) was obtained from Sigma Aldrich (St. Louis, MO, USA). Optical cutting temperature (OCT) compound was purchased from Leica (Wetzlar, Germany). Arcturus CapSure Macro LCM Caps and PEN membrane frame slides were purchased from Life Technologies (Foster City, CA, USA). Laser-capture microdissection apparatus: Arcturus^XT^ (Life Technologies, Foster City, CA, USA). Cryostat microtome: CM1950 (Leica, Wetzlar, Germany). Ammonium bicarbonate, dithiothreitol (DTT), iodoacetamide (IAA), trifluoroethonal (TFE) and formic acid (FA) were purchased from Sigma Aldrich (St. Louis, MO, USA). Sequencing grade trypsin was obtained from Promega (Madison, WI, USA). Ammonium formate was purchased from Thermo Fisher Scientific (Rockford, IL, USA). HPLC grade acetonitrile (CH_3_CN) was obtained from Thermo Fisher Scientific (Rockford, IL, USA). Isotopically labeled peptide with 99.8% purity: APGLTQALNTK (^13^C_6_, ^15^N_2_), and unlabeled peptide with the same sequence with 99.0% purity: APGLTQALNTK, were purchased from GenScript (Nanjing, China).

### 3.2. Animals

All procedures and use of animals were reviewed and approved by the Institutional Animal Care and Use Committee (IACUC) of Sun Yat-Sen University (Guangzhou, China; project identification code, IACUC-2013-1204; date of approval, 13 December 2013). Three young adult male Sprague Dawley rats weighing 250–300 g were obtained from Sun Yat-Sen University, and allowed free access to food and water before all procedures.

### 3.3. Intraluminal Middle Cerebral Artery Occlusion (MCAO) Model

Rats were weighed and placed in an ether jar until they were immobilized (*n* = 3), and anesthetized with 30% chloral hydrate in normal saline (intraperitoneally), which was supplemented as necessary during the procedure. Body temperature was monitored and maintained within normal limits with a heating pad. A midline neck incision was made and the soft tissue pulled apart. The right common carotid artery (RCCA) was carefully dissected free from the surrounding nerves (without harming the vagal nerve) and a ligature was made using 5–0 string. The right external carotid artery (RECA) is then separated and a second knot made. Next, the right internal carotid artery (RICA) is isolated and a knot prepared with a 5–0 string. After obtaining good view of the right internal carotid artery (RICA) and the right pterygopalatine artery (RPA), both arteries were clipped, using a microvascular clip. A small hole was cut in the RCCA before it was bifurcated into the RECA and the RICA. A monofilament made of 8.0 nylon (with a diameter of 0.26 mm, 40 mm in length, Beijing Cinontech Co., Ltd., Beijing, China) coated with silicon hardener mixture (with a diameter of 0.36 ± 0.02 mm) is then introduced into the RICA, until it stopped at the clip. The clipped arteries were opened while the filament was inserted into the RICA to occlude the origin of the RMCA in the circle of Willis. The third knot on the RICA was closed to fix the filament in position. The wound was closed with a surgical suture. Animals were put in a heated cage for four hours and neurological deficits were evaluated, and then the animals were sacrificed and brain tissues were collected.

### 3.4. Brain Tissue Collection and Preparation

Four hours after MCA occlusion, rats were anesthetized and perfused with 1× Phosphate Buffered Saline (PBS) (*n* = 3). Then, animals were sacrificed and brain tissue was collected after 4 h of ischemia. Slices of fresh brain tissue, 2 mm in thickness, were stained with 1% TTC to evaluate the size and extent of ischemic infarction in each rat. The rest of the tissue was embedded with OCT compound and frozen at −80 °C immediately. Fresh-frozen and OCT compound-embedded brain samples were sectioned (20 µm) on a cryostat (Leica, CM1950). Each section was placed onto PEN membrane frame slides (Life Technologies, Foster City, CA, USA), followed by H&E staining and LCM.

Sectioned tissue slides were immediately fixed in 70% ethanol for 15 s, placed in deionized water for 30 s to remove OCT and rehydrate the tissue, placed in Mayer’s Hematoxylin for 45 s to stain nuclei, placed in deionized water for 15 s to remove excess hematoxylin, placed in Scott’s Tap Water for 15 s to change the hue of hematoxylin, placed in 70% ethanol for 15 s, and then placed in Eosin Y for 3 s to stain cytoplasm. Then, H&E-stained slides were dehydrated in 95% ethanol for 30 s twice, followed by dehydration in 100% ethanol for 30 s twice. Ethanol was then removed through a 3 min bath in xylene three times.

After the tissue was air-dried, both infarcted and intact areas were microdissected with an Arcturus^XT^ system. The standard LCM protocol was followed [25]. Briefly, the tissue was cut using an ultraviolet laser with 100% transmission. Specific areas were collected onto CapSure Macro LCM Cap with the size of ~10 mm^2^ for each area.

### 3.5. On-Film Digestion

The film was carefully peeled off from the top of each cap and put into an Eppendorf tube. Microdissected cells along with the film were lysed in 50 μL of trifluoroethanol (TFE)/50 mM ammonium bicarbonate (1:1, *v*:*v*) and then sonicated in a water bath for 20 min [26,27]. Samples were reduced with dithiothreitol (10 mM) at 50 °C for 15 min followed by alkylation with iodoacetamine (20 mM) in the dark at room temperature for 30 min. The buffer was adjusted to 250 μL and tryptic digestion performed at 37 °C overnight on a shaker. The film was picked out before the samples were dried in a speedvac concentrator (Thermo SAVANT SPD1010, ThermoSavant, Holbrook, NY, USA). The digests were resuspended in 0.1% formic acid. Each sample was spiked with 0.2 ng/μL APGLTQALNTK* and APGLTQALNTK before liquid chromatography/mass spectrometry (LC–MS/MS) analysis.

### 3.6. Selection of Unique Peptides, Reference Peptides and Q1/Q3 Transition

Unique peptides for eight proteins were selected from the database of PeptideAltas and NIST Libraries of Peptide Tandem Mass Spectra Global Protein Machine. Skyline software (version 2.6, MacCoss Lab, University of Washington, Seattle, WA, USA) was used for the peptide selection. The criteria of unique peptides were as follows: (1) the length of peptides is more than 7 amino acids; (2) peptides containing methionine or tryptophan residues are excluded; (3) in peptide sequences, asparagine followed by glycine or proline is avoided; (4) no N-terminal glutamine residues in peptide sequences are selected; (5) uniqueness of selected peptides are confirmed by searching against Uniprot rat database [28].

The reference peptides (APGLTQALNTK* and APGLTQALNTK) were chosen due to their strong MRM transition signals, suitable elution time and low variability in peak area, which have been verified in another study [12].

Three MS1/MS2 transition ion pairs were selected to verify a unique peptide, three of which were applied to confirm a protein. Multiple injections of on-film digests were performed to match relative intensities of target ion pair transition signals with those of MRM transition signals observed previously in ion trap MS/MS spectra obtained from the database. Reproducibility of transition signals between different runs was also examined to eliminate the false positive rates of proteins. Collision energy and declustering potential were calculated based on the following empirical equation, which is recommended by Skyline [29]:


For doubly-charged peptides, CE = 0.057 × *m*/*z* − 4.265 (1)

For triply-charged peptides, CE = 0.031 × *m*/*z* + 7.082 (2)

DP = 0.0729 × *m*/*z* + 31.117 (3)

MRM acquisition methods were performed with fragment ion-specific tuned Collision Energy (CE) voltages and scheduled retention time. The dynamic MRM option was used for all data acquisition with a target cycle time of 1 s, with a minimum and maximum dwell time of 17 and 333 ms (60 maximum concurrent MRMs) and a 300-s MRM detection window for MRM transitions.

### 3.7. LC-Multiple Reaction Monitoring (MRM) MS Analysis

A Shimadzu UFLC XR system (Shimadzu Scientific Instruments, Columbia, MD, USA) was used to directly inject 5 μL of digest samples onto a reversed phase analytical column (ACQUITY UPLC BEH C18 1.7 μm 3.0 × 150 mm column). The column was mounted in an oven maintaining a temperature of 65 °C. Peptides in each injection were separated using a 0.4 mL/min flow rate and a gradient from 3% to 70% mobile phase B over a 30 min run time. Before each run, three-min equilibration was set up. Mobile phase A consisted of 5 mM ammonium formate in 0.1% *v*/*v* formic acid, and mobile phase B consisted of 5 mM ammonium formate in 90% ACN/0.1% formic acid. The gradient method was adopted based on previous work as followed (time: % B): 0.1 min: 9% B; 3 min: 10% B; 13 min: 17% B; 13.5 min: 18% B; 13.6 min: 21% B; 16.7 min: 22.5% B; 19.7 min: 26% B; 21.7 min: 31% B; 22.5 min: 38% B; 23.5 min: 70% B; 26.9 min: 70% B; 27 min: 3% B; 30 min: 3% B.

MRM measurements were performed by an API 4000™ (ESI-QQQ) mass spectrometer (AB Sciex, Foster City, CA, USA). The data was acquired and analyzed by AB Sciex’s Analyst software (version 1.6). The parameter settings during acquisition were as follows: 4500 V capillary voltage, a curtain gas flow of 65 psi (Ultra-High Purity nitrogen), ion source gas 1 flow at 55 psi, ion source gas 2 flow at 55 psi at a temperature of 550 °C, an MS operating pressure of 3.5 × 10^−5^ Torr, and Q1 and Q3 set to unit resolution (0.7 Full Width at Half Maximum).

### 3.8. Data Analysis

Prism4 (GraphPad Inc., La Jolla, CA, USA) software was used for comparisons across different data sets by ANOVA. “Mean ± SEM” was used to describe the variability within samples. *p*-values of <0.05 were considered significant.

## 4. Conclusions

In summary, LCM–LRP provides a feasible approach for targeted quantitative analysis of valuable protein samples, and is a very powerful tool of biomarker candidate screening and evaluation. In the present study, we found that protein biomarker candidates, including NEFL, INA, MAP2, MBP, PLP and CNP, are site-specific, but potential protein biomarkers, such as NEFH, GFAP, MAPT, TUBB3, NSE and UCHL1, are evenly distributed in the brain. These findings provide useful information for the design and analysis of relevant clinical studies in the future.

## Supplementary Materials

Supplementary materials can be found at http://www.mdpi.com/1422-0067/16/06/13427/s1.
