# Erratum: Tingting Lian, et al. Identification of Site-Specific Stroke Biomarker Candidates by Laser Capture Microdissection and Labeled Reference Peptide. *Int. J. Mol. Sci.* 2015, *16*, 13427–13441

**DOI:** 10.3390/ijms18040881

**Published:** 2017-04-21

**Authors:** Tingting Lian, Daixin Qu, Xu Zhao, Lixia Yu, Bing Gao

**Affiliations:** School of Bioscience and Bioengineering, South China University of Technology, Higher Education Mega Center, Guangzhou 510006, China; l.tt04@mail.scut.edu.cn (T.L.); qu.daixin@mail.scut.edu.cn (D.Q.); rogerxu8911@gmail.com (X.Z.); yulixia2015@gmail.com (L.Y.)

The authors wish to make a change to their published paper [1]. Since 2013, the authors have performed multiple experiments in many animals, including mice and rats, at both the Sun Yat-Sen University (Guangzhou, China) and China Medical University (Shenyang, China). After carefully checking the records, the authors found that experiments of these three particular animals in this study were performed in the animal facilities of the China Medical University. Section 3.2. Animals should therefore read: “The protocol in the study was approved by the Institutional Animal Care and Use Committee of the China Medical University [SYXK(Liao)20150003]. All efforts were made to ensure the animals' welfare and to lessen the number of animals used. Three young adult male Sprague Dawley rats weighing 250–300 g were allowed free access to food and water before all procedures”.

The change does not affect the scientific results. The manuscript will be updated, and the original will remain online on the article webpage.
